# Supplementary material for: Rates and drivers of aboveground carbon accumulation in global monoculture plantation forests
Source: Nat Commun. 2022 Jul 28;13:4206. doi: 10.1038/s41467-022-31380-7 (PMC9334591; doi:10.1038/s41467-022-31380-7)
Supplement: Supplementary file 1 — Supplementary Information [file 41467_2022_31380_MOESM1_ESM.pdf]

SUPPLEMENTARY INFORMATION

"Rates and drivers of aboveground carbon accumulation in global monoculture plantation forests"

Jacob J. Bukoski, Susan C. Cook-Patton, Cyril Melikov, Hongyi (Stella) Ban, Jessica C. Liu,

Elizabeth D. Goldman, Nancy L. Harris, Matthew D. Potts

Supplementary Methods

Dataset compilation

We systematically reviewed the literature to identify studies reporting data on biomass and carbon stocks in monoculture plantations. The literature search was performed on 19 April 2017 and was part of a larger effort to collect data on biomass accumulation associated with expanding forest cover more generally[1]. Studies published since 1975 were identified on Web of Science using the keyword search terms: TOPIC: (biomass OR carbon OR agb OR recover* OR accumulat*) AND (forest) AND (restorat* OR reforest* OR afforest* OR plantation* OR agroforest* OR secondary*). We reviewed the abstracts and titles of the 11,370 peer-reviewed studies that the search initially returned to identify studies focused on re-establishing tree cover (N ~ 5,464), which we further constrained to those that quantified biomass or carbon stocks (N ~ 1,400). Finally, of these ~1,400 studies, we identified 640 studies that quantified biomass stocks in forest plantations, comprising our final list of candidate studies.

We then reviewed each of these 640 studies to determine whether the study was to be included in our database. To be included, the study had to report i) empirical measures of biomass or carbon in the aboveground pool; ii) age of the plantation at the time of field measurements; and iii) a latitude and longitude pair or sufficient geographic detail from which geographic coordinates could be obtained. We focused exclusively on carbon in aboveground biomass because accurate estimates of changes in soil organic carbon stocks require pre-post measurements that are not readily available in the literature. Similarly, belowground biomass data were sparsely reported, may be unreliable due to inconsistent field sampling, and are believed to account for a small proportion of total biomass in plantation systems[2]. Prior reviews have found that up to 62% of root biomass data is unreliable due to unverifiable sampling methods[3]. We consequently did not collect information on belowground biomass stocks. Additionally, we did not collect data on understory vegetation biomass as it is a minor component of total biomass in monoculture plantation systems. Given the importance of management practices on plantation tree growth, we collected information on planting density, rotation length, site preparation, fertilization, irrigation, thinning, and vegetation control, in addition to biomass (carbon) stocks, age, geolocation, tree crop species, and prior land use/disturbance. We collected quantitative measures where possible and later coded the qualitative data on management practices to facilitate statistical analyses (described in the main text).

We did not include downed and standing dead wood (snags) as these pools comprised a relatively small percentage of stand biomass. These pools were also likely removed from the systems to reallocate growing space to living trees. The variation in our dataset captures some of the effects of density dependent self-thinning and self-pruning, but we did not address these phenomena directly as they likely had small overall effects on stand-level biomass accumulation and testing for these effects would be complicated by management (e.g.,

precommercial thinning). This dataset also does not capture carbon outcomes of failed plantations or the effects of stand-replacing disturbances. Given the large financial investments in monoculture plantations, failure rates are unlikely to be high and plantations are likely sited following risk-tolerance studies that minimize these risks of failure. These effects may be a greater challenge for studies of natural regeneration, which have highly variable outcomes in stocking and regeneration rates in space and time.

Incorporation of large datasets

In addition to the results of the literature search, we included two large compilations of aboveground carbon stocks in planted forests. The first was returned by our literature search[4], whereas the second was not returned by the search but was found at a later date[5]. We elected to include it as it filled a key geographic gap in our dataset (the Eurasian region, in particular Russia).

The first study reported biomass data across ~1,700 plots from 323 planted forest sites across China, accounting for a substantial portion of our final dataset[4]. Although others have elected to exclude the portion of this dataset corresponding to naturally regenerated forests from their analyses, we elected to retain the data for three reasons: i) China represents a very large proportion of the globe's planted forests, ii) plotting of biomass data in Chinese planted forests from this study vs. those of others did not suggest the potential introduction of bias, and iii) inclusion of the data substantially increased the number of observations in our database. To avoid duplicates in data, we ensured that observations were not already included in other studies, matched site codes where appropriate, and filtered the dataset to only monospecific plantations. Unfortunately, management information was not available for these data, and we were unable to include them in our analyses of drivers of variation in aboveground carbon accumulation.

The second study reported approximately 1,400 measurements of forest structure and aboveground biomass (carbon) from almost 300 sites across Eurasia. After reviewing the studies returned from our literature search, the Eurasian region was lacking data, particularly from Russia. The 2020 Forest Resources Assessment of the United Nations Food and Agriculture Organization estimates that roughly 20% of global planted forests exist in Russia and this was therefore a key geographic region to obtain additional data. We consequently elected to include the data reported in the compiled database. When we could obtain studies of the original papers in English (n = 28), we verified all data included in our database. Ultimately, a large portion of the data were published in other languages, and we could not review most of the original sources (n = 172). Nevertheless, the database is described in an English-language publication and was compiled by researchers from the International Institute for Applied Systems Analysis (IIASA) as well as well-respected forestry departments in Russia, Ukraine, and China. IIASA is a highly respected scientific organization, and we anticipate that the data are not only reliable, but a valuable window into forest biomass studies published in languages of the Eurasian region. Given the importance of this data for our study, we ultimately elected to incorporate it into our database.

For both data compilations, we judiciously reviewed observations from young stands with anomalously high aboveground carbon values, as well as old stands with anomalously low aboveground carbon values. When we could not verify an explanation for the anomalously high or low values, we dropped the observations from our database. These anomalously low values were approximately 2% of the total observations in our database and were primarily for *Populus* plantations in China that had less than 5 Mg AGC/ha after 25 years of growth. Given that Populus is a fast-growing genus and we cannot explain the low aboveground carbon values, we elected to drop them to avoid potentially biasing our growth function parameters.

Accounting for potential spatial autocorrelation

We grouped measurements by site to account for spatial autocorrelation. The precision of site geolocation was highly variable across studies. Some studies reported specific geolocations for each plot within

a site. We harmonized the site codes to better account for autocorrelation in environmental and climatic conditions across global scales. For example, many studies used chronosequences as their experimental design, which attempt to control for environmental and climatic conditions. We therefore coded chronosequences as coming from a single site. Plots that had repeat measurements across time (e.g., measurements of biomass across multiple years) were grouped by plot. Our handling of spatial structure in the database consequently attempts to accounts for correlation across observations at global scales.

Our coding of chronosequences as coming from a single "site" was to maximally account for spatial autocorrelation in the data under our mixed effects modeling framework. However, definitions of "sites" versus "plots" were highly variable in the studies we reviewed and our coding of sites may have induced bias into our analyses. To test for any potential effects in our estimated model parameters, we recoded all chronosequence plots as individual sites (rather than coding all plots in a chronosequence as coming from the same site) and re-estimated the model parameters. Comparing the 95% confidence intervals of the two sets of model parameters suggests that no statistically significant difference in our model parameters results from how we coded our sites (Supplementary Fig. 1). We therefore retained our maximal approach for accounting for spatial autocorrelation in the dataset.

Preprocessing and standardization of data

When studies empirically measured carbon concentrations in biomass and reported values of biomass carbon, we retained those values. Many studies used a default factor of 0.5 to convert aboveground biomass to aboveground biomass carbon, which we adjusted to the IPCC's default biomass to biomass carbon conversion factor of 0.47[6]. The majority of studies reported only biomass C in the tree crop without any reporting of understory vegetation. Further, plantations commonly remove understory biomass, and the relative contribution of understory vegetation to total biomass is small[1]. We therefore only collected tree crop biomass data. Similarly, most studies used allometric equations that estimated biomass across the stem, branch, twig, and foliage pools. We omitted those that reported only stem biomass given that substantial biomass may be found in the branch and twig pools but retained studies that reported only woody biomass (stem, branch and twig) given the relatively small contribution of foliage to total biomass.

One study reported ~600 observations of biomass for Eucalyptus plantations from eight sites in Brazil[7]. The study was focused directly on the influence of water, nutrients and stand uniformity on biomass production and was consequently highly relevant for our study. Eucalyptus plantations are managed on short rotation lengths in Brazil and inclusion of observation level data from this study heavily biased our data on tropical broadleaf forests towards younger ages. To reduce the influence of these data on our growth functions, we averaged observations that were i) from the same site, ii) were the same age, and iii) were managed via the same practices. Doing so reduced the total number of observations from this study from 649 observations to 85 and alleviated concerns that this study was overly influencing our Eucalyptus and tropical broadleaf growth models.

We identified one measurement with an anomalously high biomass accumulation rate that we could not explain (~40 Mg C ha^{-1} yr^{-1}). The value was greater than eight standard deviations from the mean biomass accumulation rate for that genus (Acacia) and we therefore excluded it. We also found several other points that had biomass accumulation rates greater than three standard deviations from their genus-level mean. Although this is a strong deviation, we retained them in the final dataset. Our measurements for stand ages greater than 100 years were largely dominated by one study, and the degree to which the study is representative of plantation forests is unclear. Given that time frames of less than 100 years are more representative of plantation forests and are adequate for assessing climate change mitigation actions, we filtered our database to those observations that were less than 100 years of age.

Finally, to help linearize the relationship between aboveground carbon and stand age, we square root transformed both variables (Supplementary Fig. 2). The linear relationship facilitates the driver analysis, which was examined through linear mixed effects models. Given that we were using our driver analysis to test for the effects of various covariates and not to predict or forecast expected values, transformation bias was not a concern. However, we did check to ensure statistical assumptions were met via graphical examination of the residuals for each model fit.

Database structure

In addition to assessing the representativeness of our dataset (see main text), we examined the internal structure of the data to ensure that no one plantation type or site is dominating the data. Specifically, we grouped the observations by biome, genus, and age class and characterized the number of observations, plots, and sites for each of the nine genera that we parameterized growth functions for. Across all biome, genus, and age class groupings, the maximum number of observations was 61 (mean = 5, median = 2). However, when additionally grouping by site (i.e., number of observations per biome x genus x age class x site), the maximum number of observations was 15 (mean = 2, median = 1), or less than 0.5% of our data. These number suggest that we had reasonable balance in observations across our biome, genus, and age class groupings. We have produced a table that provides these summary statistics of the data structure as a Supporting data file. Further, the dataset is publicly available, which will facilitate additional assessments of the representativeness and internal balance of the dataset.

Selection of the growth model functional form

We considered four growth models of different functional forms for modeling the accumulation of aboveground carbon in monoculture plantations: logarithmic, linearized logistic, logistic, and the Chapman-Richards growth function. Logarithmic curves are commonly used to approximate biomass (or carbon) accumulation in forests over time and, of the four functions considered, are the easiest to fit[8]. However, they do not account for the logistical growth pattern known to describe carbon accumulation in forests. The three remaining functions all model logistic growth patterns. The first (linearized logistic) is common within the forest economics literature and is commonly used due to its ease of fitting (taking the log of aboveground carbon linearizes the equation). The logistic and Chapman-Richards growth functions are nonlinear in form. Although more sensitive to nonlinear variations in data, they are more complicated to fit. The forms of the logarithmic (2), linearized logistic (3), logistic (4), and Chapman-Richards (1) growth functions are:

$$y(t) = \log_{\mathrm{e}}(t) \tag{2}$$

$$y(t) = e^{(a-b/t)} \tag{3}$$

$$y(t) = \frac{A}{\left(1+b*\mathrm{e}^{(-k*t)}\right)} \tag{4}$$

$$y(t) = A*\left(1-b*\mathrm{e}^{(-k*t)}\right)^{(1/(1-m))} \tag{1}$$

Where:

- *y(t)* is aboveground carbon in Mg C ha^{-1},
- *t* is stand age (years), and

- A, b, k, and m are parameters to be estimated from the data.

We considered several criteria in selecting which growth function to fit: ease of parameterization, accurate description of theoretical biomass accumulation patterns (i.e., logarithmic vs. logistic), and visual fit to the data through graphical residual analysis. After plotting all four model forms subsets of the data (Supplementary Fig. 3, data for temperate pines), it was clear that the logistic and Chapman-Richards growth functions outperformed the other two models. A key difference between the logistic and Chapman-Richards growth functions is that the logistic function assumes symmetric increases and decreases in growth rates around the inflection point of the curve, yet the original reasoning for this assumption is difficult to find. Given that the Chapman-Richards growth function is commonly employed, is theoretically defensible, and not overly complicated to fit, we elected to use it to model the accumulation of aboveground carbon.

Sensitivity analysis of fixing the "m" parameter

To facilitate comparison of growth functions across different plantation types, we fixed the m parameter of the Chapman-Richards function at 0.67, which produces the von Bertalanffy special case of the Chapman-Richards function. However, given the interdependence of model parameters within the Chapman-Richards curve (particularly m and k), it is important to examine how alternative values of m might influence our parameter estimates. We therefore fixed m at each of three values (0.5, 0.67, and 0.75) and ran our parameter estimation procedure and bootstrapped validation (see methods) for each plantation type and each value of m. We then used the bootstrapped root-mean-square errors to identify the "best fit" set of parameters for each model type (Supplementary Table 1). It is worth reiterating that while allowing m to vary can produce better fits to empirical data, we lose the ability to compare asymptotic stand-level aboveground carbon (A) and growth rates (k) across models.

Several key insights emerge from this comparison. First, increasing values of m tend to decrease our estimates of A and increase our estimates of k. This is an expected outcome, as increasing m for a given value of k will extend the concave-up portion of the curve, and thus to achieve asymptotic aboveground carbon at a given age (i.e., achieve an empirical fit to the data), a greater growth rate (k) will be estimated from the data. Second, when plotting the curves against the data, we find relatively little adjustment in the visual fit of the curve for those plantation types with large numbers of observations (e.g., *Pinus*), but more significant shifts in the shapes of the curves for those plantation types with small numbers of observations (e.g., *Acacia*) or for longer lived species. While our sensitivity analysis provides some insight into the effect of fixing m on the growth model parameters, additional work is needed to further explore these effects. Moreover, future work would do well to consider alternative nonlinear growth functions within the forestry literature, of which there are many.

Supplementary Discussion

FAMD of plant trait data

Our plant trait FAMD reduced the trait data to two axes, which accounted for 52.4% of the variance in the trait data. The first axis (32.6% of variation) primarily represented the leaf type, nitrogen fixing capacity, and leaf phenology data, whereas the second axis (19.8% of variation) overwhelmingly represented the wood density data (Supplementary Fig. 4a). Leaf compoundness did not contribute substantially to either axis. As expected, we identified strong separation of species across the broadleaved vs. needleleaved categories of leaf type, deciduous and evergreen categories of leaf phenology, and N-fixing vs. non-N-fixing categories (Supplementary Fig. 4b). Distinct clustering is seen across the four categorical traits (leaf type, leaf phenology, leaf compoundness and

nitrogen fixation capacity), whereas the spread of species within these groupings (i.e., the diagonal spread of grey points) is primarily driven by differences in wood density (Supplementary Fig. 4b).

The effect of planting density on aboveground carbon

An unexpected result that warrants additional discussion is the non-significance of "planting density" in our Full Model 2 (see the "Drivers of aboveground carbon accumulation rates" section). This result can potentially be explained by several factors. First, it is important to note that this driver was planting density (i.e., at t = 0) rather than actual stand density at the time of field inventory, and there may be minor differences between the two due to either density-dependent mortality (i.e., self-thinning) or management-driven thinning. Second, most plantations in our dataset were managed for production of harvested wood products. It is therefore likely that planting spacing was optimized to maximally capture growing space across studies, and understocked plantations were likely poorly represented in our data. Lastly, fully stocked plantations are known to exhibit strong size-density tradeoffs (which underpin well-established silvicultural tools such as Reineke's Stand Density Index). Assuming that plantations have been planted to maximally occupy growing space, it is likely that the effect of low-density plantings on aboveground carbon are partially offset by greater mean stem diameters at the stand-level. Fully teasing out this relationship would require data on mean stand diameter, which we were unable to collect consistently across studies. Nevertheless, exploratory analyses of the data suggest that when fixing both genus and age (e.g., filtering to all *Pinus* plantations of 20 years of age), we see some tendency towards greater aboveground carbon with increasing planting density.

Value of repeat measurements

While repeat measurements (i.e., multiple measurements made on the same stand across time) are included in our dataset, they are the minority. That is, most aboveground carbon measurements are taken at single points in time without the stand being revisited in the future. Greater collection and availability of repeat measurements would greatly improve understanding of aboveground carbon accumulation, as additional processes such as mortality can be better accounted for. Further, repeat measurements provide understanding of stand-level patterns of growth, which can improve the ability of analyses such as ours to assess the relative drivers of carbon accumulation. For those empirically estimating standing aboveground carbon in monoculture plantations, we highly recommend the use of repeat measurements, which will greatly advance our understanding of how biological, environmental, and human factors influence forest growth.

Supplementary References

1. Cook-Patton, S. C. *et al.* Mapping carbon accumulation potential from global natural forest regrowth. *Nature* **585**, (2020).
2. Bonner, M. T. L., Schmidt, S. & Shoo, L. P. A meta-analytical global comparison of aboveground biomass accumulation between tropical secondary forests and monoculture plantations. *For. Ecol. Manage.* **291**, 73–86 (2013).
3. Mokany, K., Raison, R. J. & Prokushkin, A. S. Critical analysis of root:shoot ratios in terrestrial biomes. *Glob. Chang. Biol.* **12**, 84–96 (2006).
4. Guo, Q. & Ren, H. Productivity as related to diversity and age in planted versus natural forests. *Glob. Ecol. Biogeogr.* **23**, 1461–1471 (2014).
5. Schepaschenko, D. The Forest Observation System, building a global reference dataset for remote sensing of forest biomass. *Sci. Data* 1–11 (2019) doi:10.1038/s41597-019-0196-1.
6. IPCC. *2006 IPCC Guidelines for National Greenhouse Gas Inventories*. (Institute for Global Environmental Strategies, 2006).
7. Stape, J. L. *et al.* The Brazil Eucalyptus Potential Productivity Project: Influence of water, nutrients and stand uniformity on wood production. *For. Ecol. Manage.* **259**, 1684–1694 (2010).
8. Poorter, L. *et al.* Biomass resilience of Neotropical secondary forests. *Nature* **530**, 211–214 (2016).

Supplementary Figures

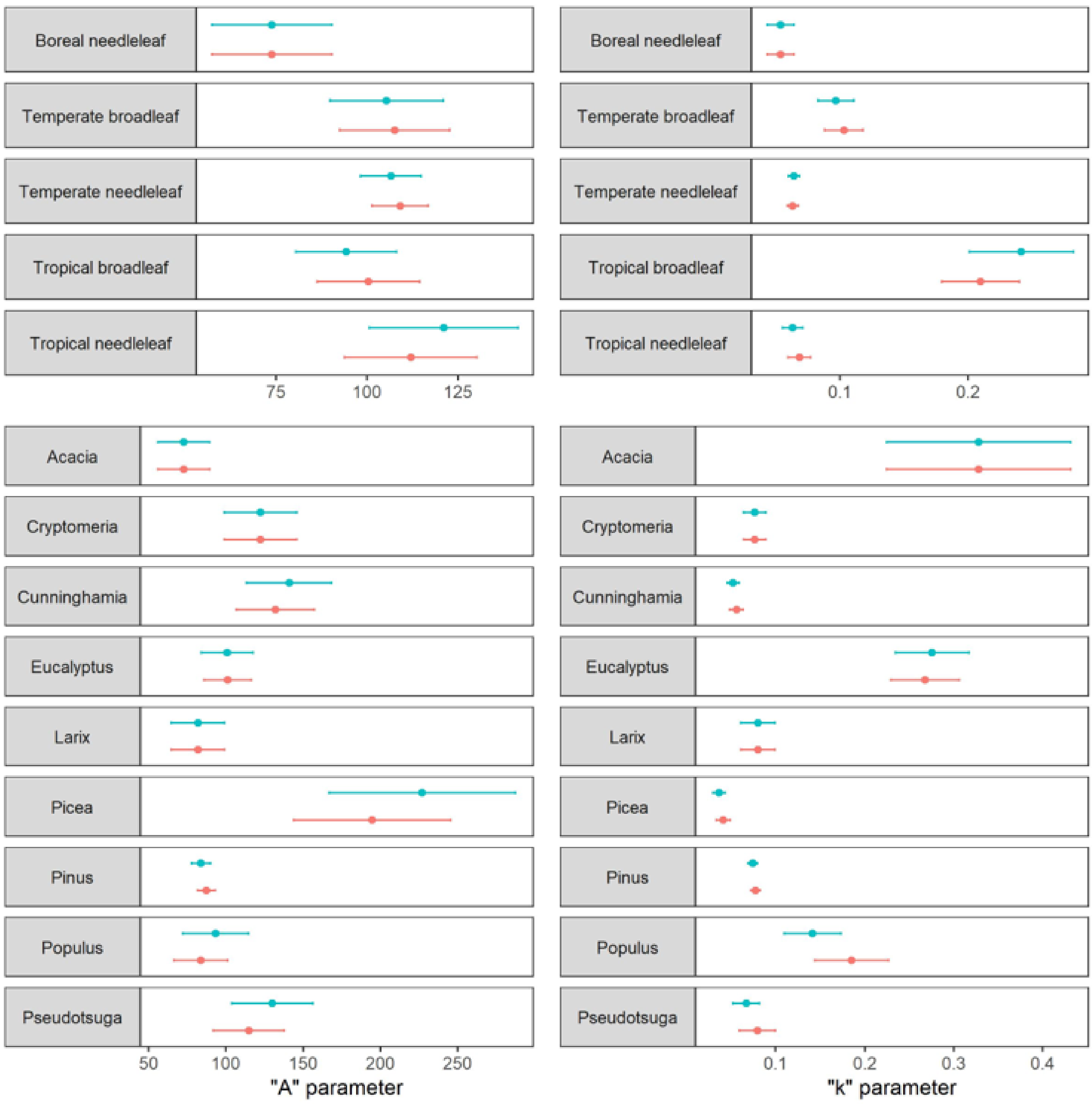

Supplementary Fig. 1 | Comparison of model parameters with chronosequence plots coded as coming from i) the same sites (blue) versus ii) individual sites (red). The points are the estimated parameter values, and the standard errors of the parameters are shown as error bars. The number of observations associated with the model parameters and standard errors vary with plant functional type / genus and are provided in Figures 2 and 3 of the main text. No statistically significant difference in the model parameters is seen when grouping plots within sites versus coding plots as individual sites.

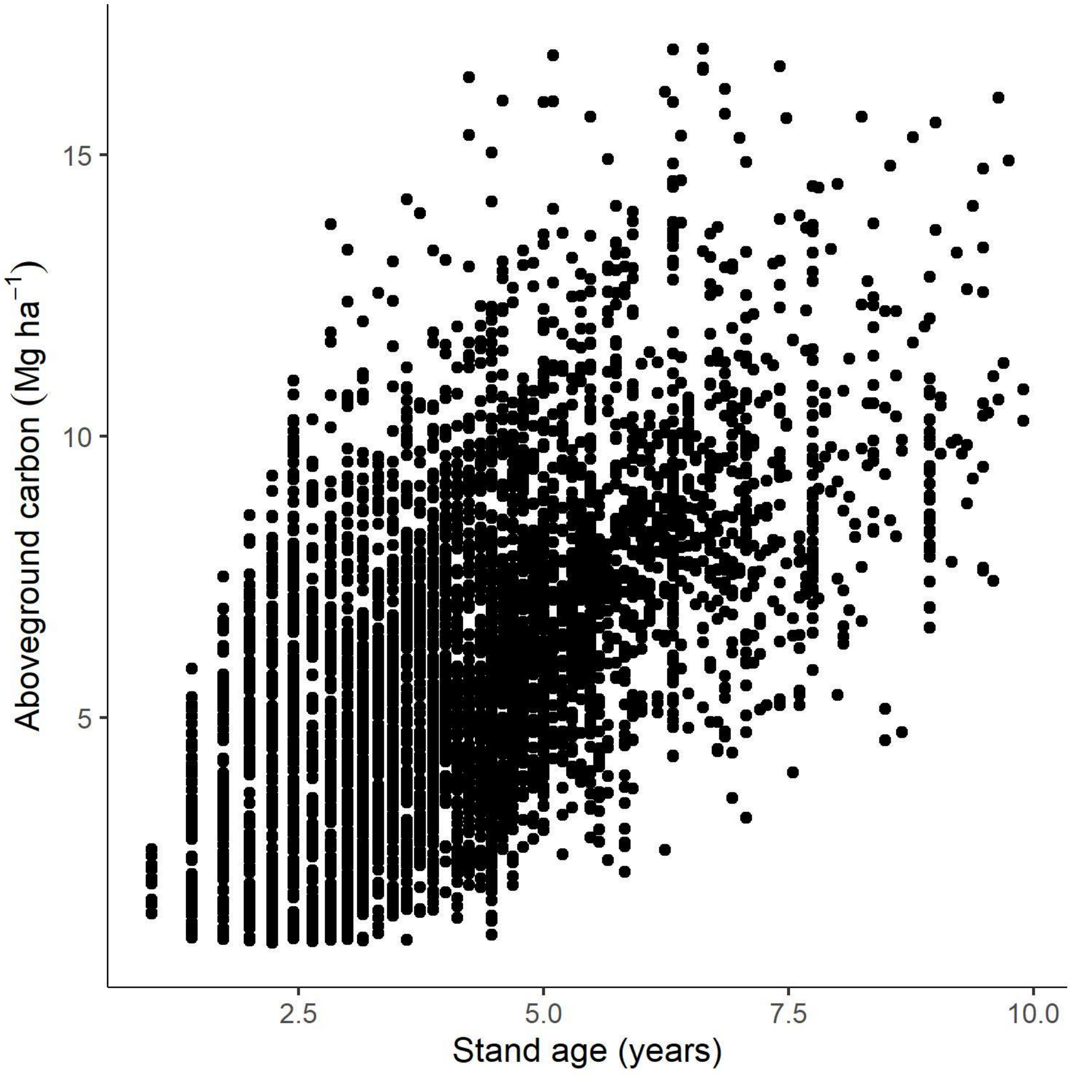

Supplementary Fig. 2 | Linearization of the aboveground carbon and stand age data via square root transformation.

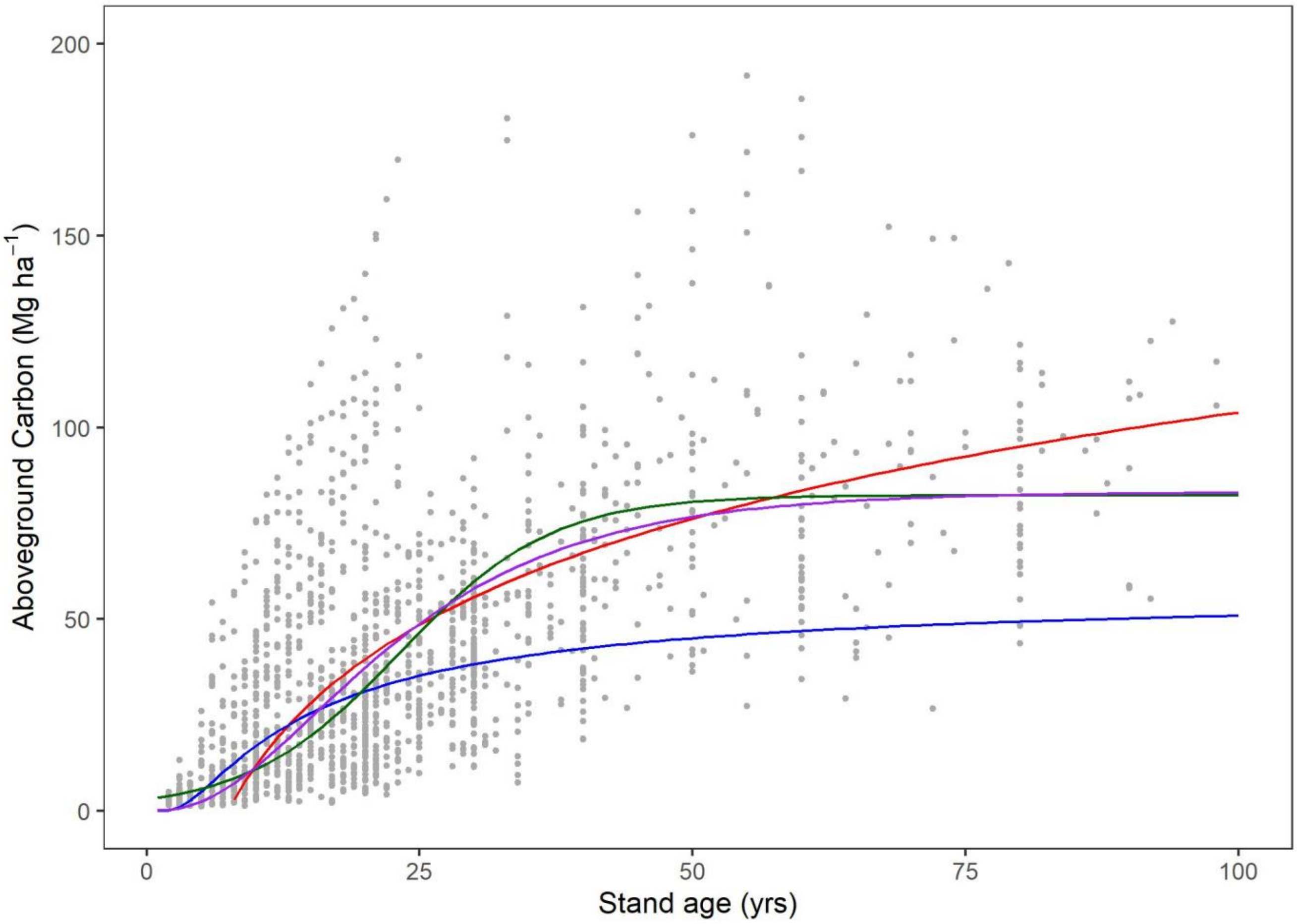

Supplementary Fig. 3 | Comparison of growth function forms. Four growth models of different functional forms fit to all temperate pine data in our database. The functions are logarithmic (red), linearized logistic (blue), logistic (green), and the Chapman-Richards growth function (purple).

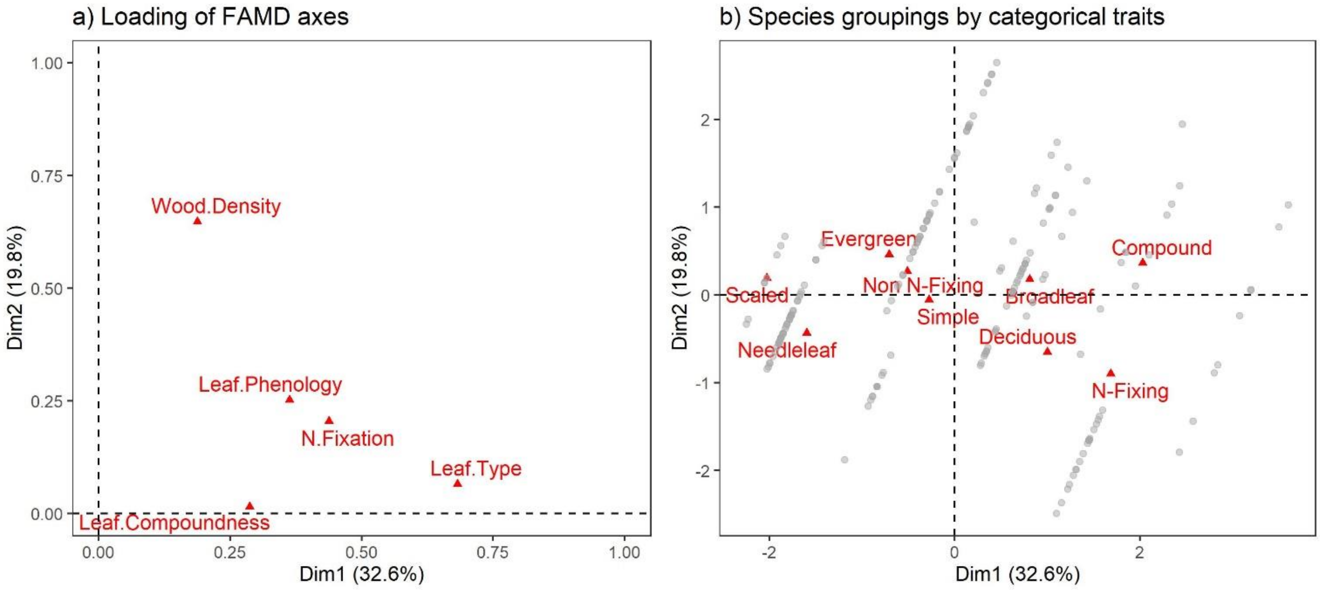

Supplementary Fig. 4 | Exploratory analysis of trait data using factor analysis of mixed data. Panel a) shows loading of the traits on the two FAMD axes, whereas panel b) shows the species-level groupings across the levels of the categorical trait data. Diagonal distribution of grey points in panel b) is driven by differences in wood density data.

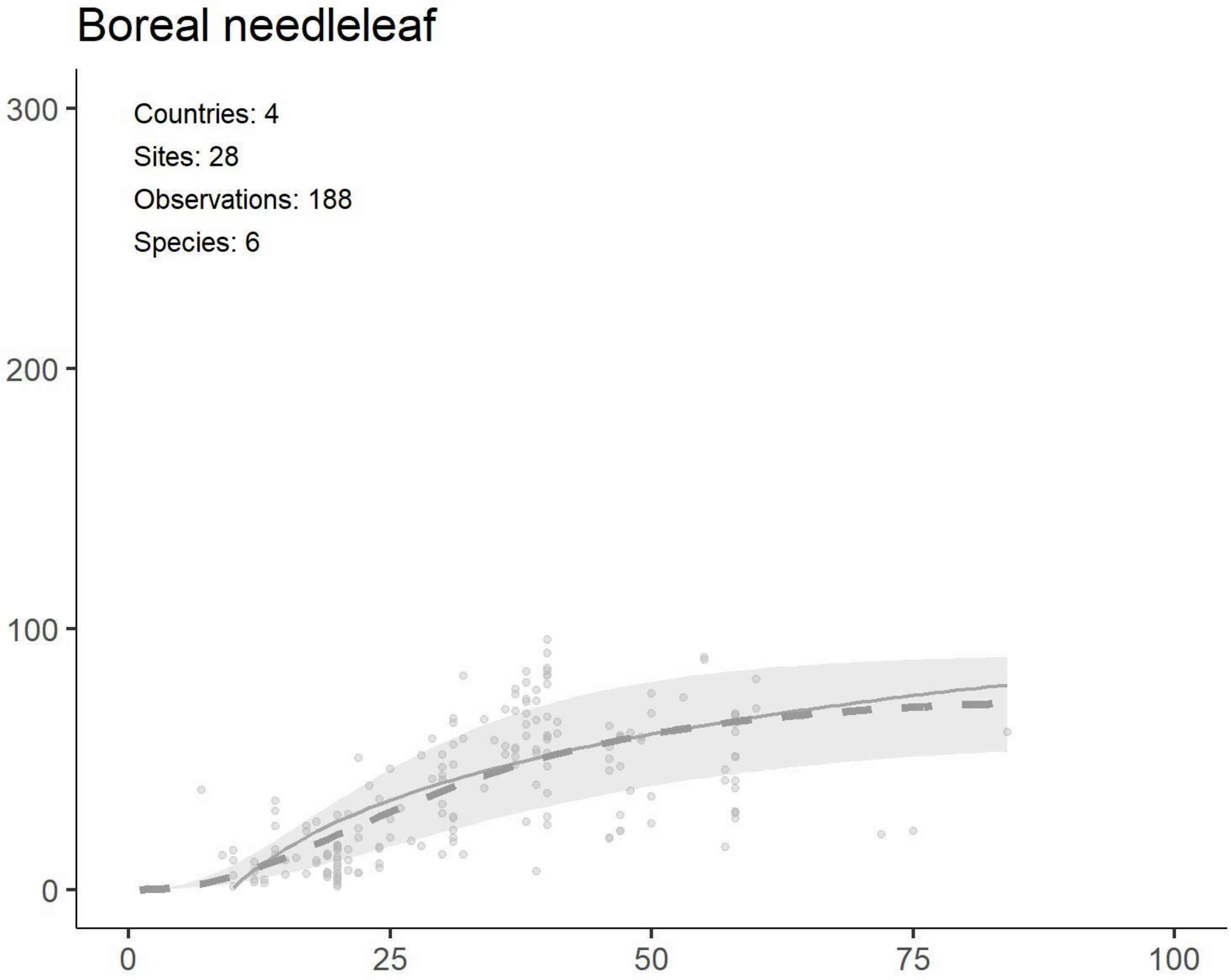

Supplementary Fig. 5 | Growth function visualization for boreal needleleaf forests. The best-fit prediction of the Chapman-Richards growth function for boreal needleleaf forests is displayed as the dashed grey line, with the shaded area around the curve corresponding to the 95% confidence interval of the prediction based on fixed effects only. The solid grey curve (for visual comparison of species level trends) is the logarithmic relationship between stand age and aboveground carbon for individual species with greater than 40 observations in the database.

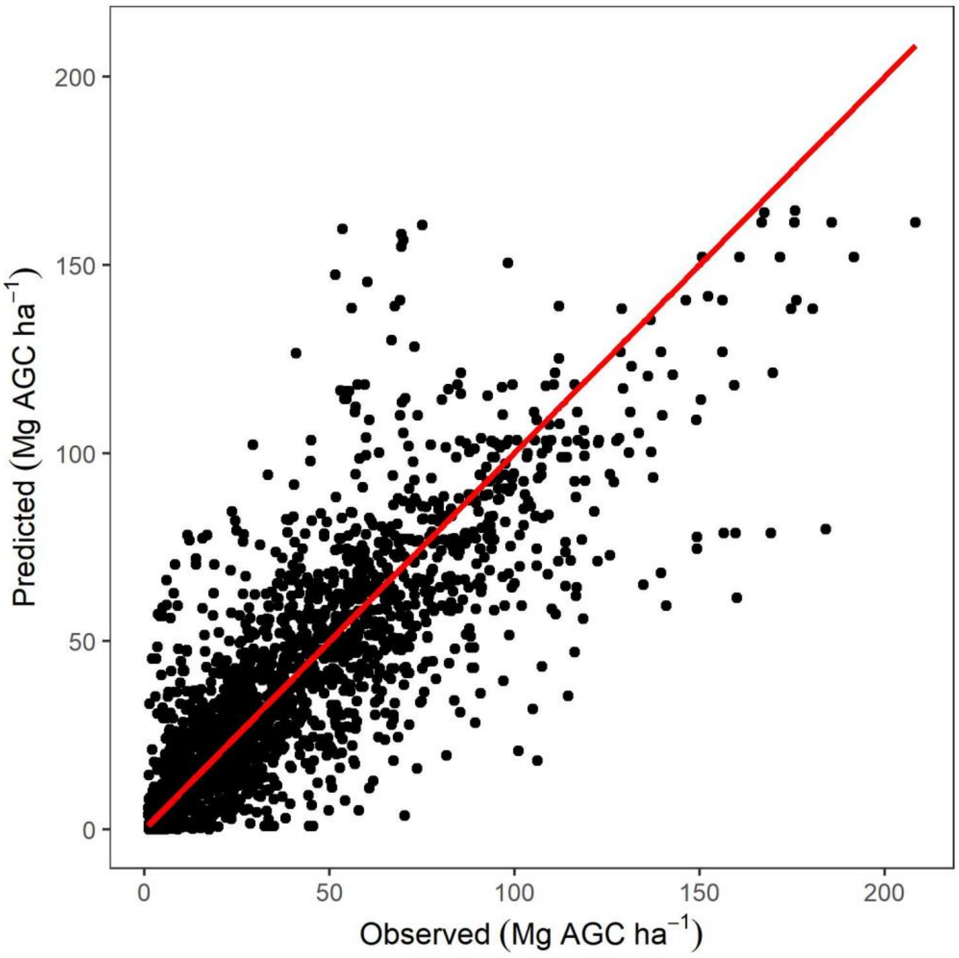

Supplementary Fig. 6 | Predicted versus observed aboveground carbon for the *Pinus* growth model parameters. Here, random effects estimated for site are incorporated into the predicted aboveground carbon values. Despite unexplained variation in the data, we see the data cluster around the 1-to-1 line (shown in red), and that, for observations in our dataset, maximum aboveground carbon stocks in *Pinus* plantations are estimated at ~164 Mg AGC ha^{-1} after incorporating site-level effects.

Supplementary Tables

Supplementary Table 1 | Potential drivers of variation in aboveground carbon accumulation rates across global monoculture plantations.

Potential Driver	Description	Levels of Variable
Genus of Tree Crop [a]	Reported genus of tree crop obtained from literature review	genus
Endemism [a]	Planting of exotic vs. endemic species	exotic, endemic
Plant Traits		
Leaf type [c]	Species-level leaf type (TRY ID 17)	broadleaf, needleleaf, scaled
Leaf compoundness [c]	Species-level leaf compoundness (TRY ID 37)	simple, compound
Leaf phenology [c]	Species-level leaf phenology (TRY ID 43)	deciduous, evergreen
Nitrogen fixation [c]	Species-level nitrogen fixation capacity (TRY ID 8)	fixing, non-fixing
Wood density [d]	Species-level wood density	continuous variable
Prior Land Use [a]	Reported prior land use obtained from literature review [a]	harvest, cropland, pasture, and fire
Management Practices [a]		
Planting density	Stem density upon planting (stems/ha)	continuous variable
Use of fertilizer	Fertilizer used in plantation	yes / no
Use of irrigation	Irrigation used in plantation	yes / no
Use of weeding	Competing vegetation controlled	yes / no
Use of thinning	Plantation thinned	yes / no
Biome [b]	Terrestrial Ecoregions of the World Map	biome type

[a] *Reported in the database compiled for this study.*
[b] *Olson et al., 2001; Dinerstein et al., 2017*
[c] *TRY Plant Trait Database*
[d] *Global Wood Density Database*

Supplementary Table 2 | Parameter estimates of the Chapman-Richards growth functions. All parameters were estimated with the *m* parameter fixed at 0.67, which helps facilitate comparison of *A* and *k* parameters across models. N corresponds to the number of observations used to estimate the growth function parameters.

**Model**	**N**	**Parameter Estimates**		**RMSE**	**Normalized RMSE** [a]
		***A* (S.E.)**	***k* (S.E.)**		
Plant Functional Types					
Boreal Needleleaf	188	73.9 (8.4)	0.054 (0.005)	38.2	1.02
Temperate Broadleaf	449	105.4 (7.9)	0.097 (0.007)	67.3	1.46
Temperate Needleleaf	2,170	106.5 (4.2)	0.064 (0.002)	64.7	1.38
Tropical Broadleaf	733	94.3 (7.1)	0.242 (0.021)	62.9	1.17
Tropical Needleleaf	612	121.0 (10.4)	0.063 (0.004)	73.9	1.53
Genera					
Acacia	115	72.8 (8.5)	0.328 (0.053)	41.4	0.74
Cryptomeria	123	122.5 (11.9)	0.076 (0.006)	71.9	1.03
Cunninghamia	461	141.0 (13.9)	0.052 (0.003)	91.6	2.13
Eucalyptus	411	100.9 (8.6)	0.276 (0.021)	62.7	1.29
Larix	221	82.0 (8.8)	0.080 (0.010)	43.0	0.92
Picea	291	227.1 (30.8)	0.036 (0.003)	122.1	2.58
Pinus	2,018	84.0 (3.1)	0.074 (0.003)	51.0	1.26
Populus	167	93.6 (10.8)	0.141 (0.016)	63.4	1.47
Pseudotsuga	195	130.1 (13.4)	0.067 (0.008)	75.6	0.96

[a] *We normalized RMSE values using mean within-group AGC (e.g., mean Pinus AGC). RMSE was calculated for each model using a 15:85% validation to training data split, bootstrapped a total of 25 times. The reported RMSE and normalized RMSE values are the averages across all 25 runs.*

Supplementary Table 3 | Chapman-Richards parameters derived for different values of *m*. Chapman-Richards parameters estimated for the fourteen different plantation types examined in our study. Three sets of parameters are presented for each plantation type, with *A* and *k* estimated empirically and *m* fixed at one of three values (0.50, 0.67, and 0.75). The set of parameters with the lowest root mean square error is recommended as the "best fit" model and is indicated in bold font. S.E. = standard errors of the parameters, n = number of bootstrapped validation runs, RMSE is root mean-square error, and nRMSE is normalized root mean square error.

Plantation Type	m	A	A (S.E.)	k	k (S.E.)	n	RMSE	nRMSE
Acacia	**0.50**	**74.9**	**9.2**	**0.262**	**0.052**	**25**	**39.1**	**0.70**
Acacia	0.67	72.8	8.5	0.328	0.053	25	41.4	0.74
Acacia	0.75	70.6	8.0	0.390	0.056	25	41.3	0.74
**Boreal needleleaf**	**0.50**	**73.1**	**8.7**	**0.040**	**0.006**	**25**	**32.1**	**0.86**
Boreal needleleaf	0.67	73.9	8.4	0.054	0.005	25	38.2	1.02
Boreal needleleaf	0.75	69.6	7.0	0.066	0.006	25	37.4	1.00
Cryptomeria	**0.50**	**143.5**	**14.7**	**0.049**	**0.005**	**25**	**67.5**	**0.96**
Cryptomeria	0.67	122.5	11.9	0.076	0.006	25	71.9	1.03
Cryptomeria	0.75	114.7	11.3	0.097	0.008	25	69.8	1.00
Cunninghamia	**0.50**	**155.6**	**17.9**	**0.034**	**0.003**	**25**	**70.0**	**1.62**
Cunninghamia	0.67	141.0	13.9	0.052	0.003	25	91.6	2.13
Cunninghamia	0.75	122.6	11.3	0.069	0.004	22	85.7	1.99
Eucalyptus	0.50	129.6	11.8	0.161	0.014	25	60.9	1.25
Eucalyptus	0.67	100.9	8.6	0.276	0.021	25	62.7	1.29
Eucalyptus	**0.75**	**90.1**	**7.4**	**0.370**	**0.028**	**25**	**59.9**	**1.23**
Larix	**0.50**	**87.6**	**10.6**	**0.057**	**0.009**	**25**	**39.1**	**0.84**
Larix	0.67	82.0	8.8	0.080	0.010	25	43.0	0.92
Larix	0.75	78.3	8.0	0.096	0.010	25	43.7	0.94
Picea	**0.50**	**260.2**	**40.3**	**0.020**	**0.003**	**24**	**97.6**	**2.06**
Picea	0.67	227.1	30.8	0.036	0.003	25	122.1	2.58
Picea	0.75	203.4	24.9	0.048	0.004	25	112.5	2.38
Pinus	**0.50**	**95.6**	**4.1**	**0.049**	**0.002**	**25**	**46.4**	**1.15**
Pinus	0.67	84.0	3.1	0.074	0.003	25	51.0	1.26
Pinus	0.75	78.9	2.8	0.093	0.003	13	50.3	1.25
Populus	**0.50**	**114.1**	**16.9**	**0.087**	**0.013**	**25**	**56.7**	**1.31**
Populus	0.67	93.6	10.8	0.141	0.016	25	63.4	1.47
Populus	0.75	99.7	11.6	0.159	0.017	25	63.5	1.47
Pseudotsuga	**0.50**	**123.3**	**14.2**	**0.060**	**0.010**	**25**	**64.4**	**0.82**
Pseudotsuga	0.67	130.1	13.4	0.067	0.008	25	75.6	0.96
Pseudotsuga	0.75	118.3	11.2	0.086	0.009	25	74.0	0.94
**Temperate broadleaf**	**0.50**	**107.4**	**8.7**	**0.069**	**0.006**	**25**	**56.2**	**1.22**
Temperate broadleaf	0.67	105.4	7.9	0.097	0.007	25	67.3	1.46
Temperate broadleaf	0.75	95.3	6.6	0.127	0.009	25	63.9	1.38
**Temperate needleleaf**	**0.50**	**124.6**	**5.8**	**0.041**	**0.002**	**25**	**62.0**	**1.33**
Temperate needleleaf	0.67	106.5	4.2	0.064	0.002	25	64.7	1.38
Temperate needleleaf*	0.75	101.2	3.8	0.078	0.002	12	64.5	1.40
**Tropical broadleaf**	**0.50**	**93.6**	**7.1**	**0.173**	**0.018**	**25**	**55.1**	**1.02**

Tropical broadleaf	0.67	94.3	7.1	0.242	0.021	25	62.9	1.17
Tropical broadleaf	0.75	89.6	6.6	0.298	0.026	25	63.2	1.17
**Tropical needleleaf**	**0.50**	**126.8**	**11.1**	**0.042**	**0.004**	**25**	**60.0**	**1.24**
Tropical needleleaf	0.67	121.0	10.4	0.063	0.004	25	73.9	1.53
Tropical needleleaf	0.75	104.0	8.3	0.084	0.005	25	67.7	1.40

** To achieve model convergence, we dropped rapid growing Pinus observations from one site (Site ID == 3969) for the Temperate Needleleaf & m = 0.75 model.*
